# Syndecan-1 expression has prognostic significance in head and neck carcinoma

**DOI:** 10.1038/sj.bjc.6690088

**Published:** 1999-02

**Authors:** A Anttonen, M Kajanti, P Heikkilä, M Jalkanen, H Joensuu

**Affiliations:** Department of Oncology, Helsinki University Central Hospital, Helsinki, FIN-20521, Finland; Department of Pathology, Helsinki University Central Hospital, Helsinki, FIN-20521, Finland; Turku Center for Biotechnology, Turku, FIN-20521, Finland

**Keywords:** proteoglycan, squamous cell carcinoma, head and neck cancer, prognosis, syndecan

## Abstract

The syndecans are a family of cell-surface heparan sulphate proteoglycans that regulate cell behaviour by binding extracellular matrix molecules such as growth factors. The syndecan family has four members, of which syndecan-1 is the most studied and best characterized. We have studied the prognostic significance of syndecan-1 expression in squamous cell carcinoma (SCC) of the head and neck treated with surgery and post-operative radiotherapy. Paraffin-embedded tissue samples taken from 175 patients with primary SCC, followed up from 2 to 15 years after surgery, were studied for expression of syndecan-1 by immunohistochemistry. A low number (≤50%, the median value) of syndecan-1-positive tumour cells was associated with low histological grade of differentiation (*P* < 0.0001), a large primary tumour size (T1–2 vs T3–4, *P* = 0.02), positive nodal status (N0 vs N1–3, *P* = 0.0006), and high clinical stage (stage I or II vs III or IV, *P* < 0.0001). Low syndecan-1 expression was also associated with unfavourable overall survival in a univariate analysis (*P* = 0.001). In a multivariate survival analysis, the clinical stage and syndecan-1 expression were the only independent prognostic factors. We conclude that syndecan-1 is a novel prognostic factor in SCC of the head and neck treated with surgery and post-operative radiotherapy. © 1999 Cancer Research Campaign


*British Journal of Cancer* (1999) **79**(3/4), 558–564
© 1999 Cancer Research Campaign
Article no. bjoc.1998.0088

### Syndecan-1 expression has prognostic significance in head and neck carcinoma

**A Anttonen[1], M Kajanti[1], P Heikkilä[2], M Jalkanen[3] and H Joensuu[1]**

Departments of [1]Oncology and [2]Pathology, Helsinki University Central Hospital, FIN-20521 Helsinki, Finland; [3]Turku Center for Biotechnology, FIN-20521 Turku, Finland

**Summary** The syndecans are a family of cell-surface heparan sulphate proteoglycans that regulate cell behaviour by binding extracellular matrix molecules such as growth factors. The syndecan family has four members, of which syndecan-1 is the most studied and best characterized. We have studied the prognostic significance of syndecan-1 expression in squamous cell carcinoma (SCC) of the head and neck treated with surgery and post-operative radiotherapy. Paraffin-embedded tissue samples taken from 175 patients with primary SCC, followed up from 2 to 15 years after surgery, were studied for expression of syndecan-1 by immunohistochemistry. A low number (≤50%, the median value) of syndecan-1-positive tumour cells was associated with low histological grade of differentiation ($P < 0.0001$), a large primary tumour size (T1–2 vs T3–4, $P = 0.02$), positive nodal status (N0 vs N1–3, $P = 0.0006$), and high clinical stage (stage I or II vs III or IV, $P < 0.0001$). Low syndecan-1 expression was also associated with unfavourable overall survival in a univariate analysis ($P = 0.001$). In a multivariate survival analysis, the clinical stage and syndecan-1 expression were the only independent prognostic factors. We conclude that syndecan-1 is a novel prognostic factor in SCC of the head and neck treated with surgery and post-operative radiotherapy.

**Keywords:** proteoglycan; squamous cell carcinoma; head and neck cancer; prognosis; syndecan

Extracellular matrix (ECM) molecules and growth factors participate in regulation of cellular behaviour by binding to specific cell-surface receptors. Earlier studies have shown that reduced intercellular adhesion, disturbed differentiation and changes in the composition of the basement membrane influence the development of malignant tumours (Weinstein et al, 1976; Liotta et al, 1986). Heparan sulphate proteoglycans (HSPGs) are macromolecules which are present at the cell surface. They are involved in cell–cell adhesion and the interaction of cells with ECM (Ruoslahti, 1989). They are also thought to act as coreceptors for the heparin-binding growth factors (HBGFs), thus participating in regulation of cell behaviour (reviewed by Bernfield et al, 1992).

Syndecan-1 is a member of a family of cell-surface HSPGs, which consists of at least four members named syndecans 1 to 4 (Bernfield et al, 1992; Jalkanen et al, 1993). They all have homologous transmembrane and cytoplasmic domains, but their extracellular domains are different (Bernfield et al, 1992; Jalkanen et al, 1983). Syndecan-1 was originally found on the surface of normal murine mammary gland epithelial cells (Rapraeger and Bernfield, 1993), and to date it is the most studied member of the syndecan family. Syndecan-1 is suggested to act as a matrix receptor, because it binds to various ECM molecules via its heparan sulphate chains and contacts the cytoskeleton through its cytoplasmic domain (Elenius et al, 1990). Components of ECM to which syndecan-1 has been shown to bind include type I, III and V collagen (Koda et al, 1985), fibronectin (Saunders and Bernfield, 1988), thrombospondin (Sun et al, 1989), tenascin (Salmivirta et al, 1991), amphoterin (Salmivirta et al, 1992) and laminin (Salmivirta et al, 1994). Syndecan-1 also binds the basic fibroblast growth factor (bFGF) (Elenius et al, 1992).

During organogenesis and tissue formation, the expression of syndecan is strictly regulated and follows morphogenetic rather than histological boundaries and correlates with the reciprocal epithelial–mesenchymal interactions (Thesleff et al, 1988; Vainio et al, 1989). In a normal adult, syndecan-1 is mainly expressed in epithelial tissue, where the strongest expression is found in stratified epithelia at the cell–cell contacts in epidermal keratinocytes (Hayashi et al, 1987; Inki et al, 1991), which suggests that syndecan-1 is involved in intercellular adhesion of keratinocytes. In keratinocytes, syndecan-1 is localized at the cell surface, predominantly in the suprabasal cell layers, whereas the basal cell layer shows only weak expression. Small amounts of syndecan-1 have been shown to be expressed in fibroblasts (Elenius et al, 1992) and endothelial cells (Kojima et al, 1992). The role of syndecan in wound healing has also been studied, revealing increased syndecan expression in proliferating and migrating epithelial cells of the epidermis and in hair follicles and some expression on the vascular endothelial cells of granulation tissue proliferation and differentiation, suggesting that syndecan is involved in growth factor regulation (Elenius et al, 1991; Bernfield et al, 1992).

Expression of syndecan-1 has been found to be decreased in cellular transformation models. The DNA-binding protein encoded by the Wilms' tumour-suppressor gene *wt* 1 has recently been found to function as a transcriptional activator of syndecan-1 expression (Cook et al, 1997). When syndecan-1 was transfected into S115 mouse mammary tumour cells, the re-expression of syndecan-1 restored the epithelial morphology of the S115 cells (Leppä et al, 1992). Mouse skin exposed to irradiation shows loss of syndecan-1 expression, and expression switches from the granular layer to the basal and lower spinous layers after 2 weeks of

*Received 10 December 1997*
*Revised 22 June 1998*
*Accepted 13 July 1998*

*Correspondence to:* H Joensuu, Department of Oncology, Helsinki University Central Hospital, Haartmaninkatu 4, FIN-00290 Helsinki, Finland daily irradiation (Liu et al, 1997). Syndecan-1 is found by immunohistochemistry in keratinizing cells of the horn pearls in well-differentiated carcinomas, but in poorly differentiated carcinomas syndecan-1 expression may be lost (Inki et al, 1992). In premalignant lesions of stratified epithelia, syndecan-1 is lost from the basally located, atypical cell layers (Inki et al, 1991). Loss of syndecan-1 from transformed cells could be one mechanism by which tumour cells lose their attachment to each other and to the ECM and become non-responsive to the signals coming from their microenvironment (Inki et al, 1994*a*).

There are few data available on the prognostic value of syndecan expression in human cancer. The two pilot studies involving 29 and 89 invasive SCC head and neck carcinomas have suggested that reduced syndecan-1 expression is associated with poor survival in SCC of the head and neck (Inki et al, 1994*b*; Pulkkinen et al, 1997), but to the best of our knowledge syndecan-1 expression has not been shown to have independent prognostic value in any type of human cancer, including cancer of the head and neck. In the present study, we investigated syndecan-1 expression with 2 syndecan-1 antibodies against human syndecan-1 in a homogeneously treated series of 175 patients with squamous cell head and neck carcinoma with a minimum follow-up of 2 years and found syndecan-1 expression to have prognostic value that was independent of clinical stage and histological grade.

### MATERIALS AND METHODS

### Patients

We collected the clinical data on 265 patients with histologically diagnosed SCC of the head and neck who were treated with surgery and post-operative irradiation in Helsinki University Central Hospital during the period from 1975 to 1990. Ninety patients were excluded because of insufficient data or non-available paraffin-embedded tissue. The remaining 175 patients had carcinoma of the larynx ($n = 51$), the tongue ($n = 50$), the floor of the mouth ($n = 22$), the lower gum ($n = 22$), the tonsil ($n = 15$) or the hypopharynx ($n = 15$). The data collected for each patient included age, sex, primary tumour site, tumour size, cervical nodal status, overall TNM stage, histological grade, treatment and cause of death. Of the 175 patients, 21 (12%) had stage 1, 29 (17%) stage II, 81 (46%) stage III and 44 (25%) stage IV cancer, but none had distant metastases at the time of the diagnosis. The study group comprised 126 (72%) male and 49 (28%) female patients. The mean age at the time of the diagnosis was 60 (range 19–86). All patients were treated with radical surgery and post-operative split-course radiotherapy to a total dose of 66 Gy and a daily fraction size of 2.0–2.2 Gy given in five fractions a week for 9 weeks, with a 3-week rest in the middle of the treatment. The minimum follow-up time of the patients after the diagnosis was 2 years (range 2–15 years). The overall 2-year survival rate was 63% and the 5-year survival rate 43%. Staging was done according to the UICC classification (1978, Table 1).

### Histology

The analysis of syndecan-1 expression was made from formalin-fixed and paraffin-embedded tumour samples using immunohistochemistry. Histology of the tumours was reviewed by one pathologist (PH), who graded the tumours according to the WHO classification (Shamugaratnam and Sobin, 1978), except for 12

**Table 1** Site and stage distribution of 175 SCCs of the head and neck

| Primary tumour | Stage | *n* | (%) |
|---|---|---|---|
| Larynx | | | |
| | 2 | 6 | (12) |
| | 3 | 32 | (63) |
| | 4 | 13 | (25) |
| Tongue | | | |
| | 1 | 20 | (40) |
| | 2 | 11 | (22) |
| | 3 | 16 | (32) |
| | 4 | 3 | (6) |
| Oral cavity[a] | | | |
| | 2 | 9 | (20) |
| | 3 | 20 | (46) |
| | 4 | 15 | (34) |
| Tonsil | | | |
| | 1 | 1 | (7) |
| | 2 | 2 | (13) |
| | 3 | 5 | (33) |
| | 4 | 7 | (47) |
| Hypopharynx | | | |
| | 2 | 1 | (7) |
| | 3 | 8 | (53) |
| | 4 | 6 | (40) |

[a]SCCs of the lower gum or the floor of the mouth.

**Table 2** Association between syndecan-1 expression and seven clinicopathological factors in SCC of the head and neck (staining with B-B4, $n = 167$)[a]

| Factor | Percentage of syndecan-1-positive tumour cells | | |
|---|---|---|---|
| | ≤50% *n* (%) | >50% *n* (%) | *P* |
| Histological grade[b] | | | |
| Grade I | 15 (30) | 35 (70) | |
| Grade II | 30 (43) | 40 (57) | |
| Grade III | 33 (94) | 2 (6) | <0.0001 |
| Stage | | | |
| 1–2 | 13 (27) | 35 (73) | |
| 3–4 | 75 (63) | 44 (37) | <0.0001 |
| Nodal status | | | |
| N0 | 38 (41) | 55 (59) | |
| N1–3 | 50 (68) | 24 (32) | 0.0006 |
| Tumour size | | | |
| T1–2 | 37 (44) | 47 (56) | |
| T3–4 | 51 (61) | 32 (39) | 0.02 |
| Karnofsky's performance status | | | |
| 60–80 | 49 (52) | 46 (48) | |
| 90–100 | 39 (54) | 33 (46) | 0.74 |
| Width of the surgical resection margin[c] | | | |
| <0.5 cm | 43 (52) | 40 (48) | |
| 0.5–2.0 cm | 43 (53) | 38 (47) | 0.87 |
| Gender | | | |
| Male | 63 (53) | 57 (47) | |
| Female | 25 (53) | 22 (47) | 0.94 |

[a]Eight slides were excluded because of technical reasons. [b]Twelve carcinomas were of a non-keratinizing type and were not graded. [c]Three cases had incomplete data.

samples which were classified as non-keratinizing carcinomas and were not graded.

### Immunohistochemistry

Syndecan-1 was studied from paraffin-embedded samples by immunohistochemistry using both a mouse monoclonal antibody against human syndecan-1 (B-B4, Serotec, Oxford, UK) and a specific rat monoclonal antibody 104–9 (produced by Dr M Jalkanen's group) against human syndecan-1- derived peptide (amino acid residues 88–110). All 175 samples were successfully stained with the 104–9 antibody and 167 slides with the B-B4 antibody (8 slides were excluded because of technical reasons).

Tissue samples were cut into 5-μm sections on Vectabond slides. The slides were kept at +37°C for up to 24 h and then deparaffinized and dehydrated. The avidin–biotin immunoperoxidase method was used. Pretreated slides were incubated with 2% normal goat serum in 1% bovine serum albumin (BSA; Sigma, St. Louis, MO, USA) for 20 min at room temperature (RT), followed by B-B4 or 104–9 antibody in 0.3% BSA overnight at RT at a concentration of 1:200 (B-B4) or 1:100 (104–9). After washing twice with phosphate-buffered saline (PBS), the slides were incubated with Vectastain biotinylated anti-mouse IgG (Vector laboratories, Burlingame, CA, USA) in the case of B-B4 or biotinylated anti-rat IgG in the case of 104–9 (Vector laboratories) in 0.3% BSA for 30 min at RT. The slides were then washed twice with

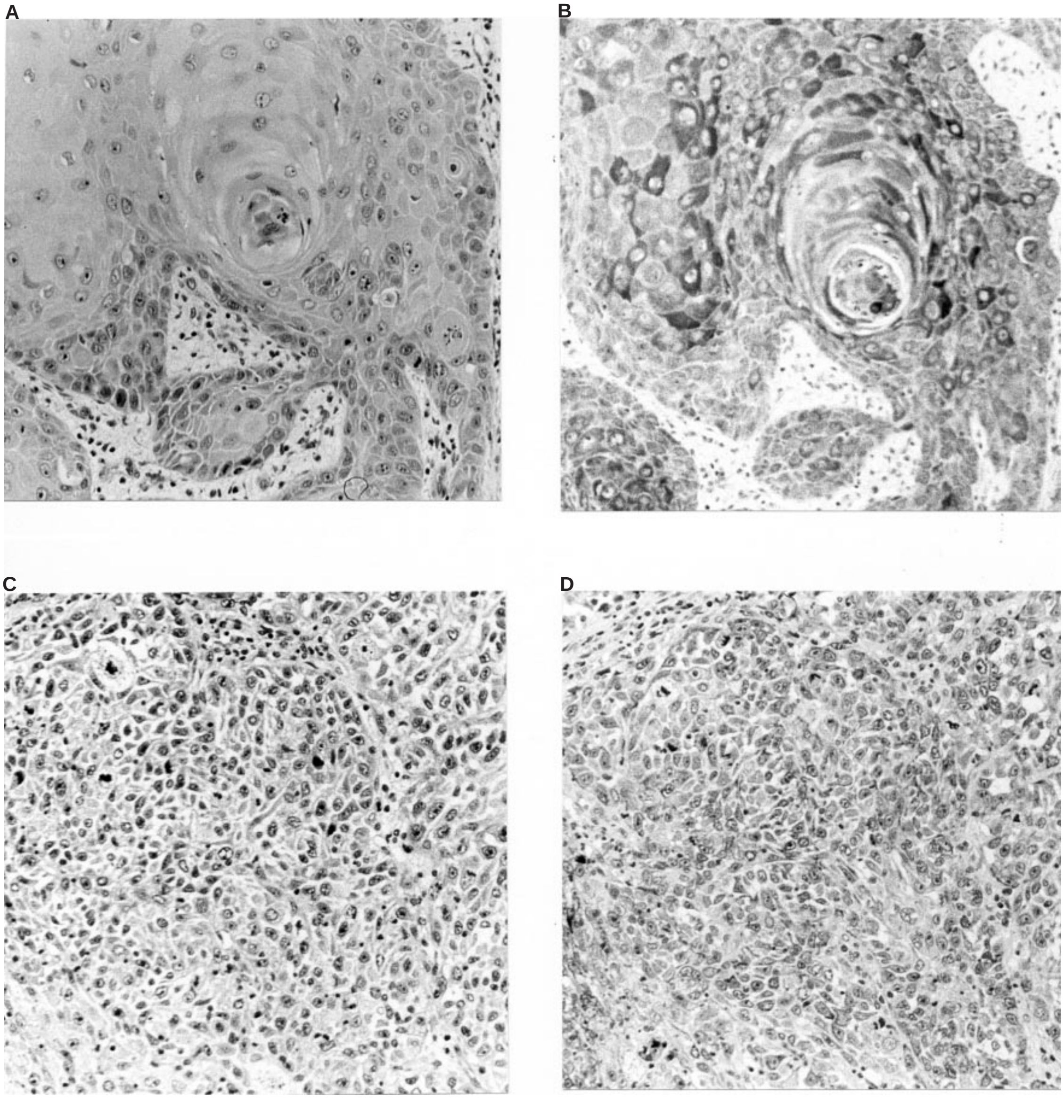

**Figure 1** Haematoxylin and eosin-stained sections (**A** and **C**) from SCCs of the head and neck, and same sections stained for syndecan-1 by immunohistochemistry (stained with 104–9 antibody (**B** and **D**). Well-differentiated SCC of the tongue (**A**), in which 100% of the tumour cells show at least some positivity in staining for syndecan-1, although the staining intensity varies markedly (**B**). Poorly differentiated SCC of the larynx (**C**), where none of the tumour cells are positive for syndecan-1 (**D**). All magnifications ×360

*© Cancer Research Campaign 1999*

PBS, followed by avidin DH–biotinylated horseradish peroxidase mixture according to the manufacturer's instructions (Vector Laboratories) for 1 h at RT. After incubation, the slides were washed again twice with PBS, and for colour reaction, the slides were incubated with 0.02% 3-amino-9-ethylcarbazole (in *N*,*N*-dimethylformamide) and 0.1% hydrogen peroxidase in 0.05 M acetate buffer, pH 5.0 for 20 min at RT, counterstained with haematoxylin and mounted. Histologically normal skin was used as a positive control and stainings without the primary antibody were used as negative controls.

Immunoreactivity of syndecan-1 was assessed visually. First, the percentage of syndecan-1-positive tumour cells was calculated from at least five representative fields per slide (Olympus Optical Company, Tokyo, Japan, ×10 objective, diameter 2.6 mm, area 5.31 $mm^2$). Then, the mean percentage of positive cells per field was calculated. One representative slide was assessed per case. Forty slides in both staining groups were reassessed by two of the investigators (AA and PH) in order to study the inter-observer variability. The inter-observer variability turned out to be low between the two observers. The kappa coefficients were 0.62 (B-B4) and 0.65 (104–9), which shows a good agreement between the observers. Assessment of syndecan expression was done blindly without any knowledge of the clinical or survival data.

### Statistical analysis

Statistical analyses were done by using a BMDP computer program (BMDP, Statistical software, University of California Press, LA, USA). Cumulative survival was estimated with the product-limit method. The Mantel–Cox's test was used for comparison of survival between groups. Frequency tables were analysed with the Chi-square test. The relative importance of prognostic factors was analysed with Cox's proportional hazard regression analysis (BMDP 2L). All *P*-values are 2-tailed.

### RESULTS

The median percentage of syndecan-1-expressing cancer cells was 50% when staining was performed with the B-B4 antibody. Only in two cases (1%) were all cancer cells positive for syndecan-1, and only ten (6%) tumours showed entirely negative staining. The results were similar independent of site, and 58%, 49%, and 43% of larynx, tongue and oral cavity carcinomas, respectively, had ≤50% of cells that stained positively for syndecan-1.

The percentage of tumour cells positive for syndecan-1 (B-B4) was >50% more often in well- or moderately well-differentiated cancers than in the poorly differentiated ones ($P < 0.0001$, Table 2). A strong (>50%) syndecan-1 expression was also associated with a low stage ($P < 0.0001$), lack of lymph node metastases ($P = 0.0006$) and a small primary tumour size ($P = 0.02$). No association between syndecan-1 expression and preoperative Karnofsky's performance status, gender or the width of the surgical resection margin was found (Table 2).

When the series was stained for syndecan-1 with the 104–9 antibody instead of B-B4, in 50 (29%) cases the percentage of syndecan-1-positive tumour cells was 80% or less, in 21 (12%) cases from 81% to 90% of the cells were positive, in 14 (8%) cases almost all cancer cells (about 95%) were positive and in 90 (51%) practically all cancer cells expressed syndecan-1. As, in this staining, in the majority of cases almost all cancer cells were positive for syndecan-1, we chose 80% as the cut-off value because it approximately defined the lowest quartile. Only three (2%) tumours showed entirely negative staining. Examples of staining are shown in Figure 1.

When the data obtained by 104–9 were used in the analyses, low syndecan-1 expression (≤80%) was again strongly associated with

**Table 3** Association between syndecan-1 expression and seven clinicopathological factors in SCC of the head and neck (staining with 104–9, *n* = 175)

| Factor | Percentage of syndecan-1-positive tumour cells ≤80% *n* (%) | >80% *n* (%) | *P* |
|---|---|---|---|
| Histological grade[a] | | | |
| Grade I | 7 (13) | 46 (87) | |
| Grade II | 16 (21) | 59 (79) | |
| Grade III | 21 (60) | 14 (40) | <0.0001 |
| Gender | | | |
| Male | 42 (33) | 84 (67) | |
| Female | 8 (16) | 41 (84) | 0.03 |
| Width of the surgical resection margin[b] | | | |
| <0.5 cm | 31 (35) | 57 (65) | |
| 0.5–2.0 cm | 19 (23) | 65 (77) | 0.07 |
| Karnofsky's performance status | | | |
| 60–80 | 24 (24) | 75 (76) | |
| 90–100 | 26 (34) | 50 (66) | 0.15 |
| Nodal status | | | |
| N0 | 25 (26) | 72 (74) | |
| N1–3 | 25 (32) | 53 (68) | 0.36 |
| Stage | | | |
| 1–2 | 13 (26) | 37 (74) | |
| 3–4 | 37 (30) | 88 (70) | 0.63 |
| Tumour size | | | |
| T1–2 | 24 (28) | 62 (72) | |
| T3–4 | 26 (29) | 63 (71) | 0.85 |

[a]Twelve carcinomas were of a non-keratinizing type and were not graded.
[b]Three cases had incomplete data.

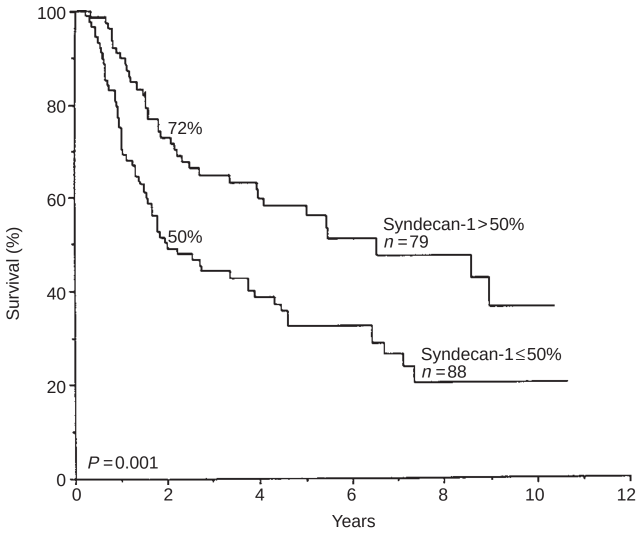

**Figure 2** Survival by syndecan-1 expression in 167 patients with SCC of the head and neck (stained with B-B4)

**Table 4** Prognostic value of nine factors in SCC of the head and neck

| Factor | *n* | Two-year survival (%) | *P*-value |
|---|---|---|---|
| Clinical stage | | | |
| St 1–2 | 48 | 86 | |
| St 3–4 | 119 | 51 | 0.0002 |
| Tumour size | | | |
| T1–2 | 84 | 76 | |
| T3–4 | 83 | 47 | 0.0002 |
| Nodal status | | | |
| N0 | 93 | 72 | |
| N1–3 | 74 | 47 | 0.0009 |
| Syndecan-1 (stained with B-B4) | | | |
| ≤50% | 88 | 50 | |
| >50% | 79 | 72 | 0.001 |
| Karnofsky's performance status | | | |
| 60–80 | 95 | 57 | |
| 90–100 | 72 | 67 | 0.006 |
| Syndecan-1 (stained with 104–9) | | | |
| ≤80% | 50 | 47 | |
| >80% | 125 | 66 | 0.02 |
| Width of the surgical resection margin[a] | | | |
| <0.5 cm | 83 | 54 | |
| 0.5–2.0 cm | 81 | 67 | 0.02 |
| Histological grade[b] | | | |
| Grade I | 50 | 71 | |
| Grade II | 70 | 57 | |
| Grade III | 35 | 53 | 0.05 |
| Gender | | | |
| Male | 120 | 61 | |
| Female | 47 | 61 | 0.50 |

[a]Three cases had incomplete data. [b]Twelve carcinomas were of a non-keratinizing type and were not graded.

a low histological grade ($P < 0.0001$, Table 3) and also with the male gender ($P = 0.03$). However, no association between syndecan-1 expression and the stage ($P = 0.63$), the nodal status ($P = 0.36$) or the primary tumour size ($P = 0.85$) was found (Table 3).

### Association of syndecan-1 expression with survival

Strong (>50%) syndecan-1 expression was associated with favourable overall survival ($P = 0.001$, B-B4 staining, Figure 2). When the percentage of syndecan-1-positive tumour cells was over 50%, the 2-year survival rate was 72% and the 5-year survival rate 56%, whereas, if the percentage of syndecan-1-positive cells was less than 50%, the corresponding rates were only 50% and 32% respectively. A similar result was found when the SCCs of the larynx (the largest single subgroup) were analysed separately: a high (>50%) syndecan-1 expression was associated with favourable overall survival ($P = 0.04$, Figure 3). Among patients with laryngeal cancer, the 2-year survival rate was 81% and the 5-year survival rate 59% if more than 50% of cancer cells stained positively for syndecan-1, but only 48% and 33%, respectively, if ≤50% of cells were positive. When the 104–9 antibody was used, a strong (>80%) syndecan-1 expression was again associated with favourable overall survival ($P = 0.02$, Table 4). If the percentage of syndecan-1-positive tumour cells was over 80%, the 2-year survival rate was 66% and, if 80% or less, only 47% (Figure 4).

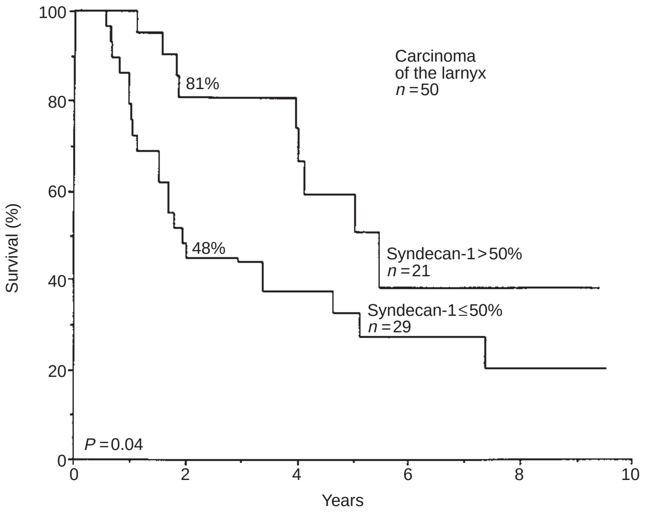

**Figure 3** Survival by syndecan-1 expression in 50 patients with SCC of the larynx (stained with B-B4)

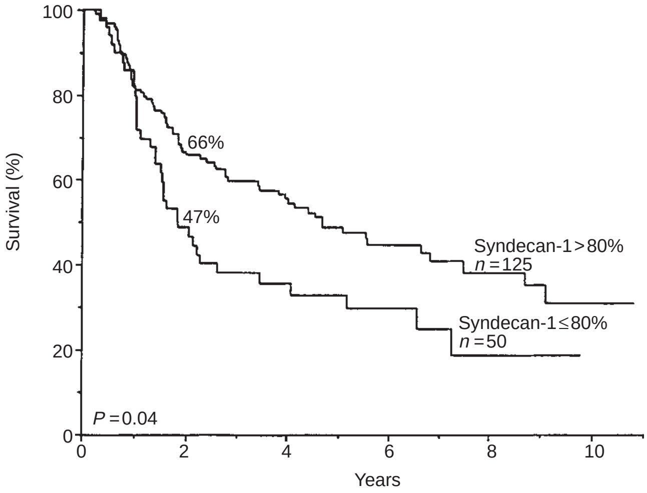

**Figure 4** Survival by syndecan-1 expression in 175 patients with SCC of the head and neck (stained with 104–9)

Several factors other than syndecan-1 expression were significantly associated with survival in the series in a univariate survival analysis (Table 4). These included the clinical stage ($P = 0.0002$), nodal status ($P = 0.0009$), tumour size ($P = 0.0002$), Karnofsky's performance status ($P = 0.006$), the width of the surgical margin ($P = 0.02$) and histological grade ($P = 0.05$). In a multivariate analysis of the entire series, the clinical stage [relative risk (RR) 2.1, 95% confidence interval (CI) 1.2–3.8], expression of syndecan-1 (B-B4) (RR 1.9, 95% CI 1.2–3.1) and possibly the width of the surgical margin (RR 1.5, 95% CI 1.0–2.4) had independent prognostic value influencing overall survival (Table 5). When we entered only the SCCs of the larynx into the model, strong syndecan-1 expression and the width of the surgical margin turned out to be the only independent factors influencing survival.

The results remained essentially similar when staining with 104–9 was used instead of B-B4 staining in a multivariate

 © Cancer Research Campaign 1999

**Table 5** Results of Cox's stepwise proportional hazard model in SCC of the head and neck

| Factor | β | s.e. | β/s.e.[a] | *P* | RR[b] (95%<1) | Step of removal |
|---|---|---|---|---|---|---|
| Clinical stage (3–4 vs 1–2) | 0.75 | 0.30 | 2.46 | <0.001 | 2.1 (1.2–3.8) | 1 |
| Syndecan-1 expression (B-B4) (≤50% vs >50%) | 0.65 | 0.24 | 2.72 | 0.016 | 1.9 (1.2–3.1) | 2 |
| Width of the surgical margin (<0.5 cm vs 0.5–2.0 cm) | 0.43 | 0.23 | 1.91 | 0.055 | 1.5 (1.0–2.4) | 3 |
| Histological grade (III vs II vs I) | | | | | NS[c] | |
| Karnofsky's status (≤80 vs >80) | | | | | NS | |
| Tumour size (T3–4 vs T1–2) | | | | | NS | |
| Nodal status (N1–3 vs N0) | | | | | NS | |

[a]β is the estimated regression coefficient of the hazard function, s.e. is the standard error and β/s.e. describes their significance. [b]RR is the relative risk, 95% confidence limits are given for the relative risk of death. [c]NS not significant.

analysis. The clinical stage (RR 2.6, 95% CI 1.5–4.4) and expression of syndecan-1 (RR 1.7, 95% CI 1.1–2.6) turned out to be the only independent prognostic factors.

### DISCUSSION

In the present study, we investigated the association of syndecan-1 expression with prognosis in SCC of the head and neck. The series consisted of 175 patients diagnosed with SCC of the head and neck and treated in a uniform fashion, and syndecan-1 expression was assessed using both a specific mouse anti-human antibody B-B4 and a rat monoclonal antibody 104–9 against human syndecan-1 by immunohistochemistry. Syndecan-1 expression (B-B4) in more than 50% of cancer cells was associated with favourable outcome in both a univariate and a multivariate survival analysis, and it was also associated with a small primary tumour size, lack of nodal metastases and, in particular, high histological grade of differentiation. The findings were similar in the entire series and in the largest subgroup studied (laryngeal cancer). Moreover, essentially similar results were obtained with the 104–9 antibody. The minor difference between the results obtained with these two antibodies can be explained by their recognition epitopes, which for B-B4 is the intact cell surface form of syndecan-1, but in the case of the 104–9 antibody, is the core protein only.

Earlier, we found a low syndecan-1 expression to be associated with poor survival, in a much smaller series consisting of only 29 patients, using a polyclonal affinity-purified rabbit antibody (anti-P117) that works only in unfixed tissue (Inki et al, 1994*b*). Although all these staining data with three different anti-syndecan-1 antibodies support the association between low syndecan-1 expression and poor survival in squamous cell head and neck cancer, it has not been established how the loss of syndecan-1 contributes to poor outcome. Normal tissue and well-differentiated SCCs show stronger syndecan-1 expression than do undifferentiated carcinomas. According to one hypothesis, syndecan-1 expression is diminished during malignant transformation and, therefore, soluble growth factors might reach more easily growth factor receptors located on the plasma membrane, resulting in more sustained stimulation of the cell growth. Cells that have lost syndecan-1 may also be more loosely bound to the extracellular matrix, which may contribute to metastasis formation.

The role of histological grading as a prognostic factor is not settled in head and neck cancer. In some studies, histological grading has been found to have prognostic value (Pera et al, 1986; Wiernik et al, 1991), while some others disagree (Bundgaard et al, 1992). In the present study, a low histological grade of differentiation was only marginally associated with unfavourable overall survival ($P = 0.05$). Although histological grading is generally considered to be a prognostic factor in head and neck cancer, it is subjective and, therefore, often difficult to reproduce (Bundgaard et al, 1992). Syndecan-1 expression may be a valuable adjunct to histological grading, because we found a high degree of uniformity in assessment of syndecan-1 expression between the two classifiers, and syndecan-1 expression appears to be associated at least as strongly with prognosis as is histological grading. Reliable methods for assessment of the biological aggressiveness of head and neck cancer are becoming more and more important because there is now evidence that the novel aggressive treatments, such as chemotherapy given concomitantly or alternating with radiotherapy (El-Sayed et al, 1996; Benasso et al, 1997), or the new radiotherapy fractionation schemes (Horiot et al, 1992), may result in improved survival.

In conclusion, decreased expression of syndecan-1 is associated with low histological grade of differentiation and poor outcome in SCC of the head and neck treated with surgery and post-operative radiotherapy. In the present series, syndecan-1 expression was the only independent prognostic factor together with the clinical stage. Hence, syndecan-1 expression appears to be a novel prognostic factor in head and neck cancer. Its role as a predictive and prognostic factor in other types of human cancer now need to be explored.

*© Cancer Research Campaign 1999* 

### ACKNOWLEDGEMENTS

We thank associate professor Seppo Sarna for advice on statistical analysis, Dr Veli-Matti Wasenius, Mrs Päivi Laurila, Mrs Päivi Tainola, Ms Päivi Heino, Ms Kati Konola and Mrs Elina Roimaa for technical advice and docent Pekka Virkkunen for help in preparing the figures. This study was supported by the Cancer Society of Finland and the Academy of Finland

### REFERENCES

Benasso M, Bonelli L, Numico G, Corvo R, Sanguineti G, Rosso R, Vitale V and Merlano M (1997) Treatment with cisplatin and fluorouracil alternating with radiation favourably affects prognosis of inoperable squamous cell carcinoma of the head and neck: results of a multivariate analysis on 273 patients. *Ann Oncol* **8**: 773–779

Bernfield M, Kokenyesi R, Kato M, Hinkes MT, Spring J, Gallo RL and Lose E (1992) Biology of syndecans. *Annu Rev Cell Biol* **8**: 365–393

Bundgaard T, Sørensen FB, Gaihede M, Søgaard H and Overgaard J (1992) Stereometric, histopathologic, flow cytometric and clinical parameters in the prognostic evaluation of 74 patients with intraoral squamous cell carcinomas. *Cancer* **70**: 1–13

Cook DM, Hinkes MT, Bernfield M and Rausher FL III (1997) Transcriptional activation of the syndecan-1 promoter by the Wilms' tumor protein WT1. *Oncogene* **13**: 1789–1799

Elenius K, Salmivirta M, Inki P, Mali M and Jalkanen M (1990) Binding of human syndecan to extracellular matrix proteins. *J Biol Chem* **29**: 17837–17843

Elenius K, Vainio S, Laato M, Salmivirta M, Thesleff I and Jalkanen M (1991) Induced expression of syndecan in healing wounds. *J Cell Biol* **114**: 585–595

Elenius K, Määttä A, Salmivirta M and Jalkanen M (1992) Growth factors induce 3T3 cells to express bFGF-binding syndecan. *J Biol Chem* **25**: 6435–6441

El-Sayed S and Nelson N (1996) Adjuvant and adjunctive chemotherapy in the management of squamous cell carcinoma of the head and neck region. A meta-analysis of prospective and randomized trials. *J Clin Oncol* **14**: 838–847

Hayashi K, Hayashi M, Jalkanen M, Firestone JH, Trelstad RL and Bernfield M (1987) Immunocytochemistry of cell surface heparan sulfate proteoglycan in mouse tissue. A light and electron microscopic study. *J Histochem Cytochem* **35**: 1079–1088

Horiot JC, Le Fur R, N'guyen T, Chenal C, Schraub S, Alfonsi S, Gardani G, Van Den Bogaert W, Danzak S, Bolla M, Van Glabbeke M and De Pauw M (1992) Hyperfractionation versus conventional fractionation in oropharyngeal carcinoma: final analysis of a randomized trial of the EORTC cooperative group of radiotherapy. *Radiother Oncol* **25**: 231–241

Inki P, Stenbäck F, Talve L and Jalkanen M (1991) Immunohistochemical localization of syndecan in mouse skin tumors induced by UV irradiation. *Am J Pathol* **139**: 1333–1340

Inki P, Kujari H and Jalkanen M (1992) Syndecan in carcinomas produced from transformed epithelial cells in nude mice. *Lab Invest* **66**: 314–323

Inki P, Larjava H, Haapasalmi K, Miettinen H, Grenman R and Jalkanen M (1994*a*) Expression of syndecan-1 is induced by differentiation and suppressed by malignant transformation of human keratinocytes. *Eur J Cell Biol* **63**: 43–51

Inki P, Joensuu H, Grenman R, Klemi P and Jalkanen M (1994*b*) Association between syndecan-1 expression and clinical outcome in squamous cell carcinoma of the head and neck. *Br J Cancer* **70**: 319–323

Jalkanen M, Elenius K and Rapraeger A (1993) Syndecan: regulator of cell morphology and growth factor action at the cell–matrix interface. *Trends Glycosci Glycotechn* **5**: 107–120

Koda J, Rapraeger A and Bernfield M (1985) Heparan sulfate proteoglycans from mouse mammary epithelial cells. Cell surface proteoglycan as a receptor for interstitial collagens. *J Biol Chem* **260**: 8157–8162

Kojima T, Scworak NW and Rosenberg RD (1992) Molecular cloning and expression of two distinct cDNAs encoding heparan sulfate proteoglycan core proteins from a rat endothelial cell line. *J Biol Chem* **267**: 4870–4877

Leppä S, Mali M, Miettinen H and Jalkanen M (1992) Syndecan expression regulates cell morphology and growth of mouse mammary epithelial tumor cells. *Proc Natl Acad Sci USA* **89**: 932–936

Liotta LA, Rao CN and Wewer UM (1986) Biochemical interactions of tumor cells with the basement membrane. *Annu Rev Biochem* **55**: 1037–1051

Liu K, Kasper M, Bierhaus A, Langer S, Peterson I, Muller M and Trott KR (1997) Differential expression of CD44 and CD44v10 proteins and syndecan in normal and irradiated mouse epidermis. *Histochem Clee Biol* **107**: 159–167

Pera E, Moreno A and Galino L (1986) Prognostic factors in laryngeal carcinoma. *Cancer* **58**: 928–934

Pulkkinen JO, Penttinen M, Jalkanen M, Klemi P and Grenman R (1997) Syndecan-1: a new prognostic marker in laryngeal cancer. *Acta Otolaryngol (Stockh)* **117**: 312–315

Rapraeger A and Bernfield M (1983) Heparan sulfate proteoglycans from mouse mammary epithelial cells. A putative membrane proteoglycan associates quantitatively with lipid vesicles. *J Biol Chem* **258**: 3632–3636

Ruoslahti E (1989) Proteoglycans in cell regulation. *J Biol Chem* **264**: 13369–13372

Salmivirta M, Elenius K, Vainio S, Hofer U, Chiquet-Erismann R, Thesleff I and Jalkanen M (1991) Syndecan from embryonic tooth mesenchyme binds tenascin. *J Biol Chem* **266**: 7733–7739

Salmivirta M, Rauvala H, Elenius K and Jalkanen M (1992) Neurite growth-promoting protein (amphoterin, p30) binds syndecan. *Exp Cell Res* **200**: 444–451

Salmivirta M, Mali M, Heino J, Hermonen J and Jalkanen M (1994) A novel laminin-binding form of syndecan-1 (cell surface proteoglycan) produced by syndecan-1 cDNA-transfected NIH-3T3 cells. *Exp Cell Res* **215**: 180–188

Saunders S and Bernfield M (1988) Cell surface proteoglycan binds mouse mammary epithelial cells to fibronectin and behaves as a receptor on interstitial matrix. *J Cell Biol* **106**: 423–430

Shanmugaratnam K and Sobin LH (eds) (1978) *Histological Typing of Upper Respiratory Tract Tumours*, pp. 14–33. WHO: Geneva

Sun X, Mosher DF and Rapraeger A (1989) Heparan sulfate-mediated binding of epithelial cell surface proteoglycan to thrombospondin. *J Biol Chem* **264**: 2885–2889

Thesleff I, Jalkanen M, Vainio S and Bernfield M (1988) Cell surface proteoglycan expression correlates with epithelial–mesenchymal interaction during tooth morphogenesis. *Dev Biol* **129**: 565–572

Vainio S, Lehtonen E, Jalkanen M, Bernfield M and Saxen L (1989) Epithelial–mesenchymal interactions regulate the stage-specific expression of a cell surface proteoglycan, in the developing kidney. *Dev Biol* **134**: 382–391

Weinstein RS, Merk FB and Alroy J (1976) The structure and function of intercellular junctions in cancer. *Adv Cancer Res* **23**: 25–33

Wiernik G, Millard PR and Haybittle JL (1991) The predictive value of histological classification into degrees of differentiation of squamous cell carcinoma of the larynx and hypopharynx compared with the survival of patients. *Histopathology* **19**: 411–417

 © Cancer Research Campaign 1999
